# Characterization of single chain antibody targets through yeast two hybrid

**DOI:** 10.1186/1472-6750-10-59

**Published:** 2010-08-22

**Authors:** Ole Vielemeyer, Clément Nizak, Ana Joaquina Jimenez, Arnaud Echard, Bruno Goud, Jacques Camonis, Jean-Christophe Rain, Franck Perez

**Affiliations:** 1 Institut Curie - Research Center, 26 rue d'Ulm, 75248 Paris cedex 05, France; 2 CNRS, UMR144, 26 rue d'Ulm, 75248 Paris, cedex 05, France; 3 U830 INSERM, 26, rue d'Ulm, 75248 Paris cedex 05, France; 4 Hybrigenics Inc., 3-5 impasse Reille, 75014 Paris, France; 5 Drexel University School of Medicine, Division of Infectious Diseases &HIV Medicine, 245N 15th Street, NCB 6th floor, Philadelphia, PA 19102, USA; 6 Université Joseph Fourier (Grenoble) Laboratoire de Spectrométrie Physique, CNRS, BP87 (UMR5588) 140 Av. de la physique, 38402 Saint Martin d'Hères, France; 7 Institut Pasteur, Membrane Traffic and Cell Division Lab, CNRS URA2582,26 rue du Dr. Roux, 75015 Paris, France

## Abstract

**Background:**

Due to their unique ability to bind their targets with high fidelity, antibodies are used widely not only in biomedical research, but also in many clinical applications. Recombinant antibodies, including single chain variable fragments (scFv), are gaining momentum because they allow powerful *in vitro *selection and manipulation without loss of function. Regardless of the ultimate application or type of antibody used, precise understanding of the interaction between the antibody's binding site and its specific target epitope(s) is of great importance. However, such data is frequently difficult to obtain.

**Results:**

We describe an approach that allows detailed characterization of a given antibody's target(s) using the yeast two-hybrid system. Several recombinant scFv were used as bait and screened against highly complex cDNA libraries. Systematic sequencing of all retained clones and statistical analysis allowed efficient ranking of the prey fragments. Multiple alignment of the obtained cDNA fragments provided a selected interacting domain (SID), efficiently narrowing the epitope-containing region.

Interactions between antibodies and their respective targets were characterized for several scFv. For AA2 and ROF7, two conformation-specific sensors that exclusively bind the activated forms of the small GTPases Rab6 and Rab1 respectively, only fragments expressing the entire target protein's core region were retained. This strongly suggested interaction with a non-linear epitope. For two other scFv, TA10 and SF9, which recognize the large proteins giantin and non-muscle myosin IIA, respectively, precise antibody-binding regions within the target were defined. Finally, for some antibodies, secondary targets within and across species could be revealed.

**Conclusions:**

Our method, utilizing the yeast two-hybrid technology and scFv as bait, is a simple yet powerful approach for the detailed characterization of antibody targets. It allows precise domain mapping for linear epitopes, confirmation of non-linear epitopes for conformational sensors, and detection of secondary binding partners. This approach may thus prove to be an elegant and rapid method for the target characterization of newly obtained scFv antibodies. It may be considered prior to any research application and particularly before any use of such recombinant antibodies in clinical medicine.

## Background

Because of their unique ability to recognize target antigens with extremely high fidelity, antibodies remain an essential tool in fundamental biomedical research and in clinical diagnostic testing. In addition, they represent one of the most promising novel therapeutic options, in particular in the field of cancer treatment. Worldwide, more than a thousand clinical trials have been completed and almost as many are currently underway using monoclonal antibodies as pharmaceuticals http://www.clinicaltrials.gov. Traditionally, monoclonal antibodies have been produced from hybridoma cell lines in a laborious process involving animal experimentation and screening large numbers of clones. More recently, recombinant monoclonal antibodies, which add an additional level of flexibility, have also become a viable treatment option for various human diseases [1-9]. Several alternatives to the classic *in vivo *selection of antibodies have been developed and refined (for summary see [10]). One of the most prominent technique is antibody phage display, which allows *in vitro *selection of single-chain variable fragment (scFv) antibodies from complex phage or phagemid libraries [11]. In this approach, which has been utilized successfully in many laboratories, animal work is obviated entirely (for review see [12,13]). It has been shown that scFv can even be manufactured for clinical use [14]. In our own research laboratory, highly functional scFv antibodies that recognize a broad range of targets, including evolutionarily conserved proteins have been selected and characterized [15-18].

Regardless of the type of production or ultimate application, the effective use of antibodies depends on a detailed understanding of the underlying antibody-antigen interaction, i.e. knowledge about a given antibody's epitope(s). This holds true especially for clinical applications where such information would be of utmost importance to accurately predict wanted and unwanted (potentially harmful) biological effects. However, target characterization of both traditional and recombinant antibodies has been challenging. The gold standard of such epitope mapping - analysis of the crystal structure of a given antigen-antibody complex - is labor intensive and sometimes nearly impossible due to technical difficulties. Thus, often cumbersome strategies have to be developed, like systematic target protein mutagenesis or sub-fragment analysis. Examples of successful characterization of antibody targets through scanning mutagenesis and other techniques have been described elsewhere [19,20]. More recently, combinatorial peptide phage display libraries have also been used [21,22]. In the future, computer based or *in silico *combinatorial methods for epitope mapping, like Mapitope [23] might prove useful. Finally, epitope mapping has also become a powerful strategy in the process of developing new vaccines [24,25].

Here we describe an alternative approach to characterize the interaction between antibodies and their respective targets. Time- and resource-demanding crystallography or mutagenesis experiments are circumvented. Instead the yeast two-hybrid (Y2H) technology is employed. In most two-hybrid applications in the recombinant antibody field, scFv are selected from a prey library [26], Here, the system is inverted: previously characterized scFv are used as bait and screened against a comprehensive cDNA library. This new approach yielded novel, detailed data about the targets of several recombinant antibodies, including the need for tertiary antigen structure for binding of conformational sensors, mapping of precise epitopes and unveiling of secondary targets.

## Results and Discussion

### Antibody targets are characterized using the two-hybrid system with the scFv antibody as bait, a validated scoring system to remove non-specific binders, and determination of selected interacting domains

**Figure 1:**
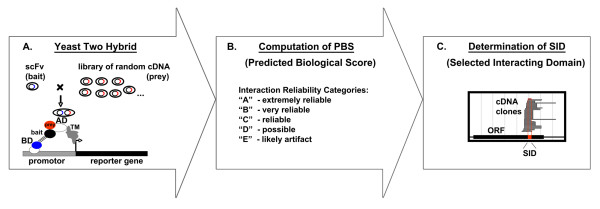
**Schematic depiction of the yeast-two-hybrid screen process using scFv as bait**. **A**. The cDNA of selected scFv is fused to the DNA binding domain of Gal4 (BD) and expressed in yeast as bait. A population of haploid yeast cells harboring short cDNA fragments fused to the activation domain of Gal4 (AD) is then used for mating. Positive clones showing interaction between bait and prey are selected through the expression of a reporter gene. (TM, Transcription Machinery). **B**. The Predicted Biological Score (PBS) is computed as described before [33] based on a statistical model of the competition for bait binding between fragments. For practical purposes, numerical numbers are converted to categories A through E as depicted. **C**. All fragments encoding for the same target ORF are aligned. This yields a selected interacting domain (SID), which narrows the area containing the antibody's binding epitope.

Investigators studying and selecting recombinant antibodies have mostly used the Y2 H technology to select antibody populations that fold correctly under the reducing conditions of the cytosol [26-31]. In such experiments, the epitope of a given antigen is defined and used as a bait to sample a diverse scFv prey library. Here, we reversed the system, using as baits known scFv antibodies that had been previously selected in phage display screens, and, as preys, proteins expressed from high-complexity libraries of randomly primed cDNA (Figure 1A).

Individual scFv antibodies were exposed to a cDNA library with full coverage, derived from either human placenta or from *Drosophila*. More than 50 million potential interactions were tested during each screen. Prey fragments of positive clones were identified using sequence analysis and comparison with GenBank databases using the BLAST algorithm [32]. Next, a Predicted Biological Score (PBS) was computed for each clone as described before [33], enabling classification according to interaction reliability (Figure 1B). This permitted ranking of the clones into categories from "A" to "D" in decreasing probability of having a specific interaction with the scFv-bait. Two additional categories were designated: "E", interacting clones, which were likely retained due to highly connected prey domains; and "F", clones that were experimentally proven Y2 H artifacts. In a third step, overlapping prey fragments originating from the same gene were designated as clusters and their translated amino acid sequences were aligned and superimposed onto the open reading frame (Figure 1C). Overlapping regions shared by all fragments were designated as "selected interacting domain" (SID), as described before [33].

### For the conformation-specific antibodies AA2 and ROF7, only large fragments are obtained, suggesting a non-linear antibody binding-site

**Figure 2:**
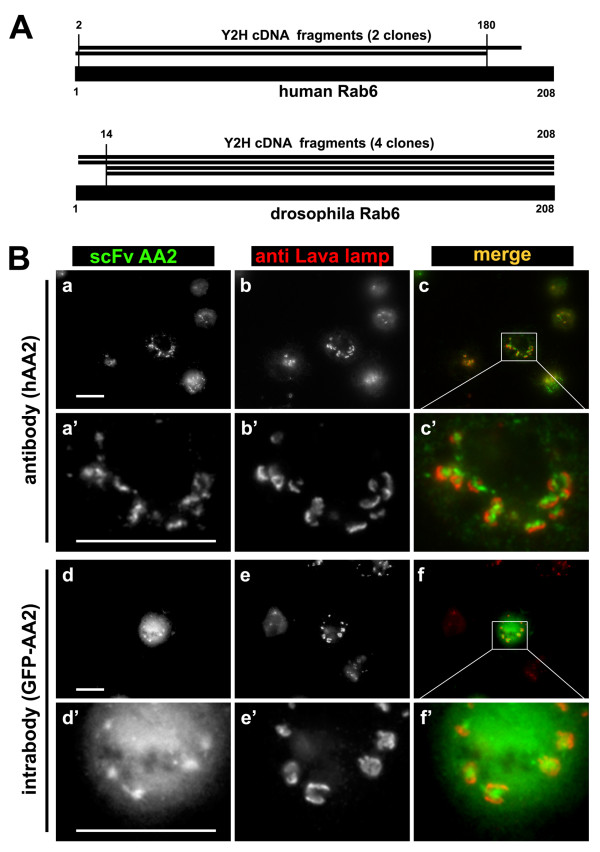
**Characterization of the scFv AA2 (anti-Rab6•GTP)**. **A**. Schematic alignment of human and drosophila Rab6 with translated sequences of their respective cDNA of the interacting clones (numbers indicate amino acid position, vertical lines project the SID onto the full length sequences). Note that in both cases the SID spans the entire core of the respective open reading frames (encoding for a protein of 208 amino acids each). **B**. AA2 recognizes the drosophila Rab6 homologue as antibody and as intrabody. **AA2 as antibody: **S2 insect cells were fixed, stained with hAA2 (a, green in c) and co-stained with anti-Lava lamp (b, red in c). Panels a', b' and c' represent a magnification of an area within a, b and c. Rab6-containing Golgi membranes are decorated with hAA2 and surrounded by Lava-lamp-positive structures. **AA2 as intrabody: **S2 cells were transfected with a plasmid expressing AA2 fused with GFP (d, green in f). Eighteen hours later cells were fixed and co-stained with anti-Lava lamp antibodies (e, red in f). Panels d', e' and f' represent a magnification of an area within d, e and f respectively. AA2 is a functional intrabody in insect cells as it stains Golgi membranes like the AA2 antibody. Stronger labeling of Golgi structures with GFP was seen in living cells (data not shown). Bar 10 μm.

In the first screen, the scFv AA2 was used as bait. AA2 is a well-described conformation-specific antibody that exclusively binds the activated (GTP bound) form of the small GTPase Rab6 [17]. From the human cDNA prey library only three specific binders were retained by Y2 H (Additional file 1). Two (67%) encoded for Rab6 (one for Rab6a and one for Rab6b, Figure 2A, top). The screen was repeated using a *Drosophila *cDNA library. Eight specific clones were retained (Additional file 2), four (50%) representing *Drosophila *Rab6 (dRab6) (Figure 2A, bottom).

The result of the *Drosophila *screen suggested that AA2, which had been selected against human Rab6a, can also recognize the fly Rab6a homologue. In our experience, polyclonal anti-Rab6 antibodies (generated by immunizing rabbits with the full-length mammalian protein) have failed to label dRab6 in immunofluorescence (unpublished observations). In contrast, and in agreement with the Y2 H data, AA2 labeling of dRab6 on Golgi membranes was seen by immunofluorescence (Figure 2B, a-c). Furthermore, analogous to what had been shown in mammalian cells [17], AA2 was also functional as an intrabody and labeled Golgi stacks in living *Drosophila *S2 cells (Figure 2B, d-f). Our Y2 H approach thus revealed that in addition to mammalian Rab6, AA2 also detects the *Drosophila *homologue. This is likely due to the fact that the three-dimensional structure of GTP-bound Rab6 remained highly conserved throughout evolution.

**Figure 3:**
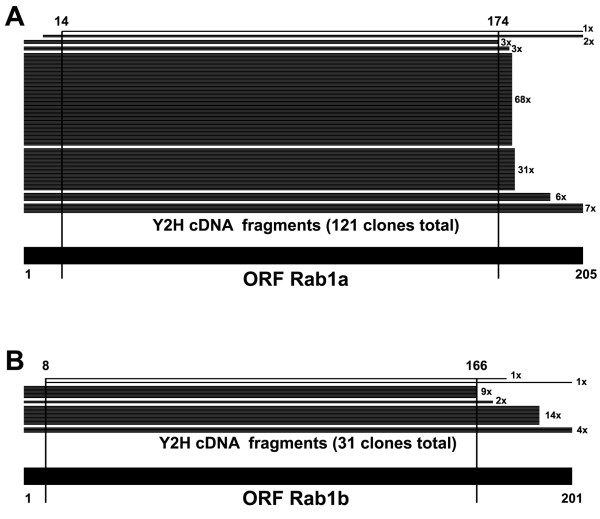
**Characterization of the scFv ROF7 (anti-Rab1•GTP)**. Schematic alignment of human Rab1a (**A**) and Rab1b (**B**) with the translated sequences of cDNA fragments found in the ROF7 screen (numbers indicate amino acid position, vertical lines project the SID onto the full length sequences). Note that in both cases the SID spans the entire core of the respective open reading frames (encoding for proteins of 205 and 201 amino acids, respectively).

In both Y2 H screens using AA2 as bait, only prey fragments spanning most of the ORF were retained (Figure 2A, and Additional files 1 and 2). Apparently, the entire core region of Rab6 had to be expressed to allow binding of this conformation-sensitive antibody. This further suggested that this method of target determination could validate non-linear epitopes. To confirm this, we next characterized the target of another recently obtained scFv, which was also observed to be conformation specific (own unpublished observations). This antibody, called ROF7, detects the small GTPase Rab1a and/or Rab1b only after their activation through GTP-loading. When the screen was performed using ROF7 as bait, a total of 191 specifically interacting clones were retained from the mammalian cDNA library. Eighty percent (152 clones) encoded for Rab1a or Rab1b, once again all encompassing the full core region of the ORF (Figure 3 and Additional file 3).

In the two Y2 H screens using conformational sensors as bait (AA2 and ROF7) and comprehensive prey libraries, only targets expressing a sizable portion of the protein (amino acids 2-178 for Rab1a, and 13-174 for Rab6a) were retained. Likely, fragments had to be large enough to allow folding into proper tertiary structure and GTP loading. The first few amino-terminal residues and the last 30 or so amino-acid long carboxy-terminal hypervariable tail are known to be dispensable for correct three-dimensional assembly of small GTPases. In fact, the crystal structure of Rab6b was solved by expressing a recombinant protein that contained only the core region of the protein (amino acids 6-181) [34]. We believe that the residues, which make up the epitopes for the binding of AA2 and ROF7, respectively, are non-adjacent in the primary amino acid sequence and only come together after correct folding of the polypeptide into tertiary structure and subsequent activation through GTP binding. Lack of reactivity of both antibodies on immunoblotting, and results from the Y2 H screens strongly supports this hypothesis.

In summary, our approach showed that for the conformation-specific sensors AA2 and ROF7 correct three-dimensional antigen structures are needed to present the antibody binding sites. It furthermore revealed that the AA2 epitope is conserved in evolution since dRab6 is recognized by the scFv.

### For the anti-giantin antibody TA10, a small binding site within the large coiled-coil protein giantin is determined

Next, we characterized the target of TA10. This scFv is a recombinant antibody directed against a very large (>350 kDa) Golgi-associated matrix protein called giantin. TA10 was originally selected via phage display using intact, purified, Golgi stacks as target [16].

**Figure 4:**
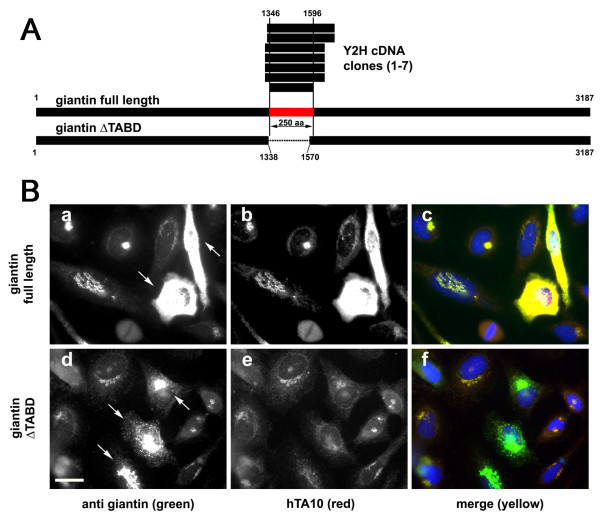
**Characterization of scFv TA10 (anti-giantin)**. **A**. Schematic alignment of full-length rat giantin with the translated sequence of the seven clones obtained from the TA10 screen (above) and the deletion construct used to confirm the localization of the interaction domain (below). Numbers indicate amino acid position relative to the full-length rat giantin sequence; horizontal lines project the SID (~28 kDa) onto the giantin sequence. This 250 amino-acid-long stretch is depicted in red. **B**. Confirmation of the ~28kDa large SID as the region which contains the TA10 epitope. HeLa cells were transfected either with full-length rat giantin (a-c) or with a giantin construct lacking identified SID (d-f). Cells were fixed 18 hours after transfection and double stained using a commercial anti-giantin antibody (green) and with hTA10 (red). Overexpression of the full-length protein was detected by hTA10 (b and c), while the staining was unchanged or even diminished in cells transfected giantin ΔTABD (e and f). Arrows indicated cells that strongly overexpress the respective constructs. Bar 10 μm.

The mammalian cDNA prey library was screened using TA10 as bait. Sixty interacting clones were retained (Additional file 4). Only one cluster, containing seven clones, obtained a high PBS ranking; they all fell within the central portion of the giantin ORF. Alignment of the seven clones defined a ~28 kDa-large SID (Figure 4A) located in the center portion of giantin. Binding of TA10 within this region of 250 amino acids was confirmed via overexpression experiments (Figure 4B).

**Figure 5:**
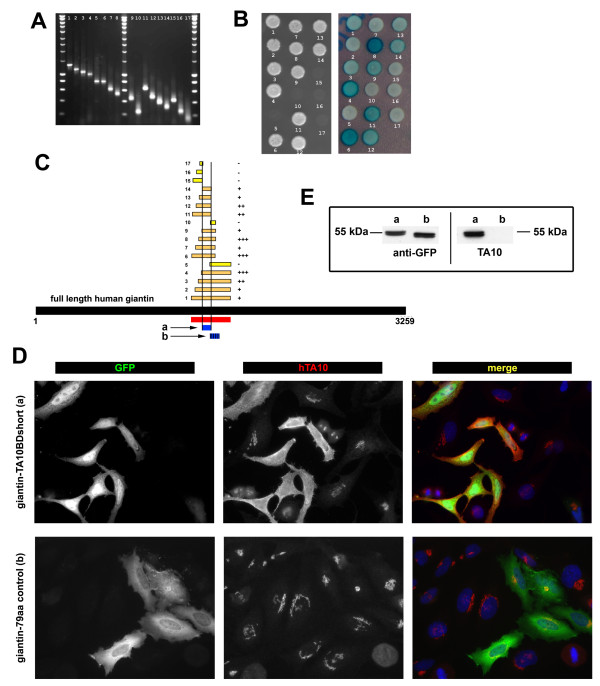
**Determination and confirmation of a precise region within the SID as the TA10 binding domain**. **A**. PCR fragments of different size and location within the smallest prey clone from the initial screen were generated and labeled from 1 to 17. **B**. The two-hybrid yeast strain containing the TA10 bait plasmid was transformed with the PCR products and a digested prey plasmid. The interactions of the prey fragment were tested by spotting in media lacking histidine (left image). A β-galactosidase qualitative observation was also done to confirm the interaction (right image). **C**. Alignment of the 17 PCR products with the human giantin amino acid sequence narrowed the putative TA10 binding domain to a 79-amino-acid long region (blue, labeled as "a") between amino acids 1462 to 1540 of the full human sequence (corresponding to amino acids 1399 to 1478 in the rat homologue). A fragment of 63 amino acids upstream of this region (labeled as "b") was used as negative control (see **D **and **E**). **D**. Confirmation using immunofluorescence. HeLa cells were transfected with a plasmid containing GFP fused to either the fragment found using the gap repair approach (called giantin-TA10BDshort, a) or the negative control (called giantin-79aa control, b). Only overexpression of the former was recognized by hTA10. **E**. Confirmation by Western blotting. The same two HeLa cell populations described in **D **were harvested and prepared for Western blotting. Only giantin-TA10BDshort (a) but not giantin-79aa control (b) was recognized by hTA10 while both were detected with anti-GFP.

TA10 is a blotting antibody and thus, in contrast to AA2 and ROF7, should detect the denatured protein at a small linear binding site. Taking advantage of the flexibility of yeast genetics, we therefore attempted to narrow the binding domain of TA10 further using a method based on gap repair. Primers were designed along the shortest of the seven prey fragments and PCR fragments were generated (Figure 5A). The Y2 H screen was then repeated, this time using the resulting small PCR products as prey (Figure 5B). In this manner, the TA10 binding region could be narrowed further to a single coiled coil domain measuring 9kDa (fragment "a" in Figure 5C). Binding of TA10 to this region was confirmed in overexpression experiments using both immunofluorescence and Western blotting (Figure 5D, E). Finally, carrying out additional biochemical and overexpression experiments we narrowed the antibody-binding region even further (within a 30 amino acid stretch, data not shown).

In summary, the Y2 H screen enabled rapid and accurate target characterization for TA10. A precise region of 79 amino acids, representing less than 2.5% of the entire protein, was determined as its binding site.

### For the non-muscle myosin IIA-targeting antibody SF9, a precise epitope within a long coiled-coil protein is determined and cross reactivity with human and other, non-human, homologues is revealed

SF9 was selected alongside TA10 in a phage display screen using purified rat liver Golgi stacks as the antigen. SF9's target, non-muscle myosin IIA, was identified using immunoblotting followed by mass spectrometry analysis [16].

**Figure 6:**
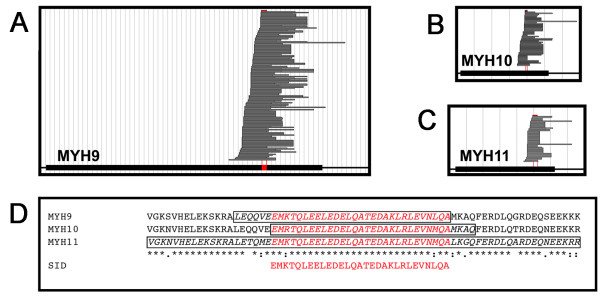
**Determination of a very precise region within myosin as the putative SF9 binding domain**. Alignment of cDNA fragments within the ORF (thick black line) of the three non-muscle myosin heavy chains. 196 cDNA fragments of MYH9 (**A**), 55 of MYH10 (**B**) and 61 of MYH11 (**C**) were found in the screen, yielding SID of 35, 33 and 70 amino acids in length, respectively (depicted in red). **B**. Sequence alignment of the three myosin-homologue SID (boxed) determines a precise 29-amino-acid long putative binding domain. Note almost complete (>93%) amino acid sequence identity for the common SID (depicted in red).

From the Y2 H screen with SF9 as bait a total of 352 specifically interacting clones were retained (Additional file 5). More than half (196 clones) encoded for non-muscle myosin IIA and their alignment allowed identification of a very small SID of only 35 amino acids (Figure 6A). An additional 116 clones represented one of two very closely related myosins: non-muscle myosin IIB and smooth-muscle myosin (55 and 61 clones, respectively). Their alignments yielded similarly precise SID (Figure 6B, C). All three SID overlapped with one another inside a conserved stretch of the tail region of the myosin protein family, further narrowing the putative binding domain for SF9 (Figure 6D).

**Figure 7:**
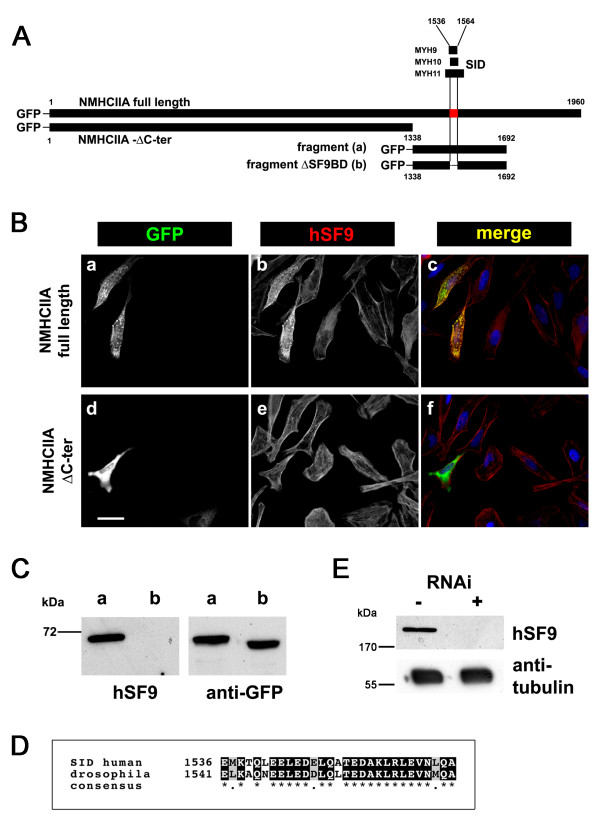
**Confirmation of the binding of SF9 to an epitope within the common SID**. **A**. Schematic alignment of the full-length human non-muscle myosin IIA heavy chain (NMHCAIIA) protein, of the three found SID and of the constructs made to confirm SF9 binding. The common (29aa long) SID is depicted in red. **B**. Analysis using immunofluorescence. HeLa cells were transfected with full-length NMHCAIIA (a-c) or the NMHCIIA-ΔC-ter fragment expressing only the first 1338 amino acids and lacking the common SID (d-f). Only the full length but not the truncated recombinant GFP-tagged protein was recognized by hSF9 (yellow in panel c versus green in panel f). Bar 10 μm. **C**. Analysis using immunoblot. HeLa cells were transfected with either a GFP-tagged NMHCIIA fragment containing the common SID (fragment, a), or a GFP-tagged NMHCIIA fragment deleted from the common SID (ΔSF9BD, b). Immunoblot analysis of total cell extracts showed that hSF9 only detected the larger fragment (lane a) while the anti-GFP antibody recognized both recombinant proteins at ~70 kDa. **D**. Sequence alignment of the 29-amino-acid long human non-muscle myosin IIA SID with the corresponding insect protein. Note the high level of similarity. **E**. Specific binding of SF9 to the drosophila protein. Total cell extract of drosophila (S2) cells incubated (+) or not (-) with RNAi to inactivate the expression of zipper (the unique myosin II homologue in *drosophila*) were analyzed by immunoblot. A specific band at ~200 kDa is present only in non treated cells. Tubulin stained by an anti α-tubulin antibody was used as loading control.

Binding of the antibody to the 29-amino-acid long common SID was confirmed in a series of overexpression experiments. GFP-tagged non-muscle myosin IIA constructs, which either included or lacked the SID (Figure 7A), were overexpressed in mammalian cells. In addition to the endogenous myosin pool, only the overexpressed full-length but not the truncated protein was recognized in immunofluorescence by SF9 (Figure 7B). Immunoblot analysis revealed that a recombinant protein containing the 29-amino-acid long SID was detected while no binding was seen with the construct lacking this short region (Figure 7C). Together, this confirmed our Y2 H results.

Non-muscle myosins are evolutionarily conserved proteins. In addition, SF9 was found to bind to a region of the protein that shares near-identical amino acid sequences between human homologues (Figure 6D). Alignment of the SID with non-muscle myosins of other species revealed a high degree of evolutionary conservation in this region even with the *Drosophila *counterpart (Figure 7D). We thus speculated that SF9 might also detect the insect form of myosin. Indeed, when using total insect cell lysates a single ca. 200-kDa-large band was detected by SF9. This signal completely disappeared after specific anti-myosin RNAi-mediated gene silencing (Figure 7E).

In summary, our Y2H-approach allowed precise characterization of the SF9 target, defining a very small (<3kDa) epitope within a ~200kDa protein. In addition, it revealed cross-reactivity with closely related proteins of the same and across species.

### Results of screens using antibodies that do not work as intrabodies

Our novel approach yielded important additional information about the targets of several distinct monoclonal antibodies. However, some variability in the strength and reliability of the data could be noted when comparing individual screens. For instance, when screening using yet another scFv, named F2C (an antibody which binds α-tubulin [16]), results were less conclusive. While a total of 380 interacting clones were obtained, only 4 encoded for α-tubulin (data not shown). Their alignment allowed narrowing of the epitope-containing region to the C-terminal portion of the protein. However, many of the remaining 376 clones were also classified as possible binders using the PBS scoring method. Nonetheless, it is unlikely that they represent secondary targets for F2C given a highly specific signal of F2C in both immunofluorescence and Western blotting [16]. Another scFv originating from the above mentioned Golgi-stack screen, called TE5 [16], was tested in our approach. While many interacting clones were retained, none could be confirmed in overexpression experiments and the TE5 target remained elusive so far (data not shown). In sharp contrast to the just mentioned examples (F2C, TE5) our ROF7- and SF9-screens not only confirmed the antibodies' targets but also gave detailed information about their respective epitopes: in each case almost 90% of clones aligned within its target(s) (see Figures 3 and 6). In agreement with the more classical use of scFv in Y2 H [26] there seems to be a direct correlation between the ability of an antibody to fold correctly under the reducing conditions of the eukaryotic (yeast or mammalian) cytosol and their use in Y2 H. While SF9, TA10, AA2 and ROF7 efficiently bind their respective targets when expressed as intrabodies, F2C is only faintly staining microtubules in living cells and TE5 never yielded specific staining as an intrabody. We thus hypothesize that the same relative efficiency for target recognition is present during the yeast two hybrid experiments. To make full use of our method, it will therefore be important to obtain good intrabodies, for example via screening of dedicated libraries [35,36] or through the use of single domain antibodies, like camelidae antibodies, that seem to be more resistant to the reducing conditions of the intracellular milieu [37].

## Conclusions

We present a novel approach, which allows in-depth characterization of antibody target(s) using the Y2 H technology. In our approach the traditional orientation of prey and bait is reversed, complex cDNA prey libraries are screened, and a robust scoring system to eliminate non-specific binders is employed. This way, critical new information about antibodies' targets can be obtained. This includes strong corroborative evidence for conformational specificity and the need for a non-linear epitope for conformational antibodies, precise mapping of small epitopes, and information about cross reactivity within and across species.

In summary, our Y2 H approach represents a powerful method for the detailed characterization of scFv/antibody targets. Given the high quantity and quality of new data that can be obtained from a simple experiment, we believe that our approach might in the future serve as an invaluable tool for the detailed target characterization of many types of antibodies.

## Methods

### Preparation of bait vectors, Y2 H cloning strategy, Y2 H screens

The plasmids pHEN2 containing the ORF for the previously described and characterized scFv (AA2, TA10, and SF9) [16,17] as well as the anti Rab1 scFv ROF7 (unpublished data), were used for sub-cloning (via use of the restriction enzymes NcoI and NotI) into a pBTM116 derived bait vector as described [38]. The generation of random-primed human placenta and *drosophila *cDNA libraries in SfiI polilynker adapted pGADGH plasmid has been described previously as well [33,39]. In such libraries, inserts have an average size of between 700 (*drosophila*) and 800 (human placenta) base pairs and the complexity reaches over 50 million clones in *E. coli*. The DNA of a maxiprep (Quiagen) prepared from *E. coli *scraped colonies was transformed into yeast strain Y187 using the classical lithium acetate protocol and plated on four hundred 140 mm Petri plates. Conditions were optimized by determining the linearity of colony forming unit (cfu) as a function of DNA concentration to avoid the transformation of individual yeast cells with multiple plasmids, because this could lead to the identification of false positive clones. After colony formation, the four hundred dishes were scraped. The resulting cells were pooled, then aliquoted and stored in glycerol 40% at -80°C as equivalent fractions of the same library. Screens were performed using a cell-to-cell mating method described previously [40]. Briefly: four hundred million prey cells were mixed with eight hundred million bait cells and spread on YDP plates for four hour to ensure mating but avoid replication of the already formed diploid cells. This ensures a complete coverage of the highly complex library because a minimum of fifty million clones is tested (10 times the primary complexity of the yeast transformed cDNA library). The genetic saturation of the screens allows statistical analysis of the results of each screen [33]. In addition, the use of the same library for many screens permits the identification of fragments that are highly connected and thus are potential "false positives" [33]. Qualitative comparison of lacZ reporter activation was done by standard X-Gal Top agar overlay assay. For details of the analysis of obtained clones see firsts paragraph "Results and Discussion" section. Lists of specifically interacting clones for each screen that are not provided in this manuscript in Additional files 1 through 5 can be requested from the corresponding author.

### Cloning of expression plasmids for confirmation of predicted epitopes

For expression of the scFv AA2 fused to the Green fluorescent protein (GFP) in insect cells the mammalian expression plasmid (pEGFP-N3NN-AA2, [17]) was digested with EcoRI and XbaI. The released fragment was then cloned into identical sites within the *drosophila *expression vector pMT/V5 (Invitrogen).

For TA10 related experiments an untagged full-length giantin expression plasmid (pSG5-GCP364) was received as a kind gift from Yoshio Misumi (Fukuoka, Japan). The mammalian expression plasmid giantin ΔTABD was generated by removing a 702-nucleotide long piece by cutting (and subsequently religating) with PmlI within the ORF of giantin of the plasmid pSG5-GCP364. For the generation of the plasmid giantin-TA10BDshort the following primers were used for PCR: Forward 5'- CCC *AAG CTT *TGT GAG CTA AAG AAG CAG CC -3' and Reverse 5'- CG*G GAT CC*T TAC TTT CCT AGG AGT GCA TC -3'. For giantin-79aa control the following primers were used for PCR: Forward 5'- CCC *AAG CTT *AGC CAG GTT TCT GTT CAG AAT -3' and Reverse 5'- CG*G GAT CC*T TAT TCG GTG CTC TCT GCA ATC TT -3'. For both PCR reactions pSG5-GCP364 was used as the template. Amplified segments were digested with HindIII and BamHI (visualized in *Italics *in the primers) and inserted into a pEGFP expression vector prepared by digestion with the same enzymes.

For experiments regarding scFv SF9 we obtained the expression plasmid containing the full-length non-muscle myosin heavy chain IIA (pTRE GFP NMHCIIA) from Robert Adelstein (National Institutes of Health Bethesda, MD, U.S.A.) via Addgene^®^. NMHCIIA-ΔC-ter was generated by digesting pTRE GFP NMHCIIA with EcoRI and SpeI, blunting with Klenow and religating, thus creating a truncated fusion protein lacking the last 620 aa of MNHCIIA. In order to remove only the 29 amino acid long putative SF9 binding domain in its C-terminal portion the following multi-step strategy was used: (i) PCR amplification of a NMHCIIA fragment ranging from the end of the putative SF9 binding domain until beyond the unique SacII site creating a KpnI site at the 5'-end using the following primers: Forward1 5'-GG*G GTA CC*A TGA AGG CCC AGT TCG AGC GG-3' (KpnI site in *Italics*), Reverse1 5'-AGG TCG GTG TTG ATC TGG TC-3'; (ii) insertion of the digested PCR product (PCR I) into the pEGFP-C1 vector (Clontech) equally digested with KpnI and SacII; (iii) a second PCR amplification (PCR II) using the same NMHCIIA-template, a forward primer before the unique EcoRI site (Forward2 5'-AAA GGG GAC TCG GAG CAC-3') and a reverse primer annealing just before the beginning of the putative SF9 binding domain again containing a KpnI site in frame (Reverse2 5'-GG*G GTA CC*C TCC ACC TGC TGC TCT AGG GC-3', KpnI site in *Italics*); (iv) digestion of both, the PCR II product as well as the pEGFP-C1 vector containing PCR I with KpnI and EcoRI and insertion of the former into the latter creating the plasmid pEGFP-fragment-ΔSF9BD (non muscle myosin heavy chain II A fragment delta SF9 binding domain). In a separate reaction the original pTRE-GFP-NMHCIIA as well as the empty plasmid pEGFP-C1 were digested with EcoRI and SacII and the 1055 bp MYH9 fragment was inserted in frame with GFP creating the plasmid pEGFP-fragment. All constructs were verified by sequencing.

### Cell cultures, immunofluorescence and immunoblotting analyses

HeLa cells were grown under standard conditions and transfected as described [16]. For immunofluorescence respective scFv (AA2, TA10, SF9) were first converted to dimeric mini-antibodies (hAA2, hTA10, hSF9) by adding a human Fc portion as described before [41]. For immunofluorescence experiments cells were then incubated with secondary anti-human antibodies (Jackson ImmunoResearch Laboratories, Westgrove, PA, USA and Molecular Probes, Eugene, OR, USA). For immunoblotting, total cell lysates of transfected HeLa cells were boiled in sample buffer and subjected to SDS-PAGE. After transfer onto nitrocellulose, membranes were blocked and then incubated with mouse anti-GFP antibodies (Roche Diagnostics GmbH, Mannheim Germany), with the humanized scFv hSF9 or with mouse anti α-tubulin antibodies (B-5-1-2, Sigma Aldrich) and revealed with ECL after incubation with respective secondary antibodies coupled with horseradish peroxidase. Transfection of the fragment constructs with a predicted molecular weight of ~70 kDa (see details in Figure 4C) were used for immunoblotting, since non-muscle myosin is present abundantly in HeLa cells and it is difficult to visualize the overexpressed protein as an additional band given the size of non-muscle myosin heavy chain (~200kDa).

Drosophila S2 and S2R cells were grown and prepared for transfection and immunofluorescence as described elsewhere [42]. Briefly, S2 cells were plated into 24-well plates (BD Falcon) at 160,000 cells/well in 1 mL growth medium (Schneider's Drosophila medium (GIBCO, supplemented with 10% heat inactivated fetal calf serum). Cells were subsequently transfected with plasmid DNA using the CaPO_4 _method [43]. The following day, transfected cells were plated on glass cover slips coated with concavalin A and after 20 minutes fixed for 5 min with 4% paraformaldehyde in PBS or with 100% methanol at -20°C. Incubation with primary and secondary antibodies of transfected or non-transfected S2 and S2R cells was performed as described above for HeLa cells.

For knock down of zipper (myosin II homologue in *drosophila*) dsRNAs were prepared as described using the following primers: left primer: 5'-CCT AAA GCC ACT GAC AAG ACG-3' right primer: 5'-CGG TAC AAG TTC GAG TCA AGC-3' creating a PCR product of 647 bp. T7 sites were added symmetrically and processed as before [42].

### Gap Repair for TA10 Epitope determination

Seventeen PCR fragments of different size were generated using as matrix the A-135 prey clone (Additional file 4). At the extremity of each fragment, 50 nucleotides corresponding to the polylinker of the prey vector were added to allow "gap repair" recombination. The transformation of the two-hybrid yeast strain containing TA10 bait plasmid with the PCR product and a digested prey plasmid resulted in the production of yeast transformants containing the bait and different prey fragments. The interactions of the prey fragment were tested by spotting in minus histidine media and by β-galactosidase qualitative observation. All fragments were aligned along the ORF of giantin.

## Authors' contributions

CN initiated the project, constructed the first plasmids and contributed to the writing of the manuscript. OV wrote the manuscript and carried out most of the characterization experiments with the help of AJJ. AE was involved in experiments using drosophila cells. BG supported the work and participated in its critical analysis. JCR carried out all yeast two hybrid experiments including molecular biology related to it. FP, JC and JCR designed the study. FP and JCR were involved in writing the manuscript, supported and directed the work. All authors read and approved the final manuscript.

## Supplementary Material

Additional file 1 **AA2 Two Hybrid Screen Results using human cDNA library**. A table listing the identity of all the hits recovered in the two-Hybrid screen using AA2 as a bait against a human cDNA library. The table presents the name and accession number of each prey (identified by alignment, see materials and methods), the nucleotide start and stop of the insert, whether it is in frame or out of frame (OOF), its sense in the prey vector and the calculated PBS score (see materials and methods). Click here for file

Additional file 2 **AA2 Two Hybrid Screen Results using drosophila cDNA library**. A table listing the identity of all the hits recovered in the two-Hybrid screen using AA2 as a bait against a drsophila cDNA library. The table presents the name and accession number of each prey (identified by alignment, see materials and methods), the nucleotide start and stop of the insert, whether it is in frame or out of frame (OOF), its sense in the prey vector and the calculated PBS score (see materials and methods). Click here for file

Additional file 3 **ROF7 Two Hybrid Screen Results using human cDNA library**. A table listing the identity of all the hits recovered in the two-Hybrid screen using ROF7 as a bait against a human cDNA library. The table presents the name and accession number of each prey (identified by alignment, see materials and methods), the nucleotide start and stop of the insert, whether it is in frame or out of frame (OOF), its sense in the prey vector and the calculated PBS score (see materials and methods). Click here for file

Additional file 4 **TA10 Two Hybrid Screen Results using human cDNA library**. A table listing the identity of all the hits recovered in the two-Hybrid screen using TA10 as a bait against a human cDNA library. The table presents the name and accession number of each prey (identified by alignment, see materials and methods), the nucleotide start and stop of the insert, whether it is in frame or out of frame (OOF), its sense in the prey vector and the calculated PBS score (see materials and methods). Click here for file

Additional file 5 **SF9 Two Hybrid Screen Results using human cDNA library**. A table listing the identity of all the hits recovered in the two-Hybrid screen using SF9 as a bait against a human cDNA library. The table presents the name and accession number of each prey (identified by alignment, see materials and methods), the nucleotide start and stop of the insert, whether it is in frame or out of frame (OOF), its sense in the prey vector and the calculated PBS score (see materials and methods). Click here for file
