# Supplementary material for: Characterization of single chain antibody targets through yeast two hybrid
Source: BMC Biotechnol. 2010 Aug 22;10:59. doi: 10.1186/1472-6750-10-59 (PMC2936416; doi:10.1186/1472-6750-10-59)
Supplement: Additional file 1 — AA2 Two Hybrid Screen Results using human cDNA library. A table listing the identity of all the hits recovered in the two-Hybrid screen using AA2 as a bait against a human cDNA library. The table presents the name and accession number of each prey (identified by alignment, see materials and methods), the nucleotide start and stop of the insert, whether it is in frame or out of frame (OOF), its sense in the prey vector and the calculated PBS score (see materials and methods). [file 1472-6750-10-59-S1.PDF]

Additional file 1: AA2 Two Hybrid Screen Results using human cDNA library

Gene Name (Best Match)	Start	Stop	Frame	Orientation	Global PBS
hRAB6A; NM_198896.1	3	537	IF	Sense	D
hRAB6B; NM_016577.2	39	616	IF	Sense	D
hSNX5; NM_152227.1	-109	743	IF	Sense	E

PBS, Predicted Biological Score
