# Supplementary material for: Characterization of single chain antibody targets through yeast two hybrid
Source: BMC Biotechnol. 2010 Aug 22;10:59. doi: 10.1186/1472-6750-10-59 (PMC2936416; doi:10.1186/1472-6750-10-59)
Supplement: Additional file 2 — AA2 Two Hybrid Screen Results using drosophila cDNA library. A table listing the identity of all the hits recovered in the two-Hybrid screen using AA2 as a bait against a drsophila cDNA library. The table presents the name and accession number of each prey (identified by alignment, see materials and methods), the nucleotide start and stop of the insert, whether it is in frame or out of frame (OOF), its sense in the prey vector and the calculated PBS score (see materials and methods). [file 1472-6750-10-59-S2.PDF]

Additional file 2: AA2 Two Hybrid Screen Results using drosophila cDNA library

Gene Name (Best Match)	Start	Stop	Frame	Orientation	Global PBS
Drosophila melanogaster - BtbVII ; GID: 24656485	1086	1911	IF	Sense	E
Drosophila melanogaster - Dlc90F ; GID: 24647918	51	ND	IF	Sense	D
Drosophila melanogaster - Hsc70-4 ; FBgn0001219	1293	1898	IF	Sense	D
Drosophila melanogaster - Rab6 ; FBgn0015797	3	834	IF	Sense	A
Drosophila melanogaster - Rab6 ; FBgn0015797	18	ND	IF	Sense	A
Drosophila melanogaster - Rab6 ; FBgn0015797	39	1015	IF	Sense	A
Drosophila melanogaster - Rab6 ; FBgn0015797	39	ND	IF	Sense	A
Drosophila melanogaster - mor ; FBgn0002783	909	2387	IF	Sense	C
Drosophila melanogaster - mor ; FBgn0002783	909	ND	IF	Sense	C

ND : no data

PBS, Predicted Biological Score
