# Supplementary material for: Characterization of single chain antibody targets through yeast two hybrid
Source: BMC Biotechnol. 2010 Aug 22;10:59. doi: 10.1186/1472-6750-10-59 (PMC2936416; doi:10.1186/1472-6750-10-59)
Supplement: Additional file 3 — ROF7 Two Hybrid Screen Results using human cDNA library. A table listing the identity of all the hits recovered in the two-Hybrid screen using ROF7 as a bait against a human cDNA library. The table presents the name and accession number of each prey (identified by alignment, see materials and methods), the nucleotide start and stop of the insert, whether it is in frame or out of frame (OOF), its sense in the prey vector and the calculated PBS score (see materials and methods). [file 1472-6750-10-59-S3.PDF]

Additional file 3: ROF7 Two Hybrid Screen Results using human cDNA library

Gene Name (Best Match)	Start	Stop	Frame	Orientation	Global PBS
CHD7 ; GID: 54112402	2538	3597	IF	Sense	D
DDX23 ; GID: 41327770	ND	2356	??	Sense	A
DDX23 ; GID: 41327770	684	1994	IF	Sense	A
DDX23 ; GID: 41327770	684	1994	IF	Sense	A
DDX23 ; GID: 41327770	684	1994	IF	Sense	A
DDX23 ; GID: 41327770	684	1994	IF	Sense	A
DDX23 ; GID: 41327770	684	1994	IF	Sense	A
DDX23 ; GID: 41327770	684	1994	IF	Sense	A
DDX23 ; GID: 41327770	684	1994	IF	Sense	A
DDX23 ; GID: 41327770	819	2388	IF	Sense	A
DDX23 ; GID: 41327770	819	2388	IF	Sense	A
DDX23 ; GID: 41327770	831	2140	IF	Sense	A
DDX23 ; GID: 41327770	834	2357	IF	Sense	A
DDX23 ; GID: 41327770	834	2357	IF	Sense	A
DDX23 ; GID: 41327770	834	2357	IF	Sense	A
DDX23 ; GID: 41327770	834	2357	IF	Sense	A
DDX23 ; GID: 41327770	834	ND	IF	Sense	A
DDX23 ; GID: 41327770	939	2378	IF	Sense	A
DDX23 ; GID: 41327770	939	2378	IF	Sense	A
DDX23 ; GID: 41327770	939	2378	IF	Sense	A
DDX23 ; GID: 41327770	939	2378	IF	Sense	A
DDX23 ; GID: 41327770	939	2378	IF	Sense	A
DDX23 ; GID: 41327770	1056	2406	IF	Sense	A
HEY-L ; GID: 105990530	33	646	IF	Sense	F
LOC342897 ; GID: 41150997	345	1308	IF	Sense	D
MMP1 ; GID: 13027798	93	805	IF	Sense	D
MYCBP2 ; GID: 126116564	7080	7772	IF	Sense	D
NUDT4 ; GID: 98985815	1433	2740	OOF2	Sense	N/A
RAB1A ; GID: 41350195	-139	523	IF	Sense	A
RAB1A ; GID: 41350195	-139	523	IF	Sense	A
RAB1A ; GID: 41350195	-139	523	IF	Sense	A
RAB1A ; GID: 41350195	-128	670	OOF2	Sense	A
RAB1A ; GID: 41350195	-125	539	OOF2	Sense	A
RAB1A ; GID: 41350195	-125	539	OOF2	Sense	A
RAB1A ; GID: 41350195	-125	539	OOF2	Sense	A
RAB1A ; GID: 41350195	-125	539	OOF2	Sense	A
RAB1A ; GID: 41350195	-125	539	OOF2	Sense	A
RAB1A ; GID: 41350195	-125	539	OOF2	Sense	A
RAB1A ; GID: 41350195	-122	640	OOF2	Sense	A
RAB1A ; GID: 41350195	-122	640	OOF2	Sense	A
RAB1A ; GID: 41350195	-119	541	OOF2	Sense	A
RAB1A ; GID: 41350195	-119	541	OOF2	Sense	A
RAB1A ; GID: 41350195	-119	538	OOF2	Sense	A

RAB1A ; GID: 41350195	-119	541	OOF2	Sense	A
RAB1A ; GID: 41350195	-119	541	OOF2	Sense	A
RAB1A ; GID: 41350195	-119	541	OOF2	Sense	A
RAB1A ; GID: 41350195	-119	541	OOF2	Sense	A
RAB1A ; GID: 41350195	-119	537	OOF2	Sense	A
RAB1A ; GID: 41350195	-119	541	OOF2	Sense	A
RAB1A ; GID: 41350195	-119	541	OOF2	Sense	A
RAB1A ; GID: 41350195	-119	541	OOF2	Sense	A
RAB1A ; GID: 41350195	-119	541	OOF2	Sense	A
RAB1A ; GID: 41350195	-119	541	OOF2	Sense	A
RAB1A ; GID: 41350195	-119	541	OOF2	Sense	A
RAB1A ; GID: 41350195	-119	538	OOF2	Sense	A
RAB1A ; GID: 41350195	-119	541	OOF2	Sense	A
RAB1A ; GID: 41350195	-119	541	OOF2	Sense	A
RAB1A ; GID: 41350195	-119	538	OOF2	A	Sense
RAB1A ; GID: 41350195	-119	541	OOF2	Sense	A
RAB1A ; GID: 41350195	-119	537	OOF2	Sense	A
RAB1A ; GID: 41350195	-119	537	OOF2	Sense	A
RAB1A ; GID: 41350195	-119	537	OOF2	Sense	A
RAB1A ; GID: 41350195	-119	537	OOF2	Sense	A
RAB1A ; GID: 41350195	-119	541	OOF2	Sense	A
RAB1A ; GID: 41350195	-119	541	OOF2	Sense	A
RAB1A ; GID: 41350195	-119	541	OOF2	Sense	A
RAB1A ; GID: 41350195	-119	537	OOF2	Sense	A
RAB1A ; GID: 41350195	-119	541	OOF2	Sense	A
RAB1A ; GID: 41350195	-119	537	OOF2	Sense	A
RAB1A ; GID: 41350195	-119	538	OOF2	Sense	A
RAB1A ; GID: 41350195	-119	537	OOF2	Sense	A
RAB1A ; GID: 41350195	-119	541	OOF2	Sense	A
RAB1A ; GID: 41350195	-119	541	OOF2	Sense	A
RAB1A ; GID: 41350195	-119	541	OOF2	Sense	A
RAB1A ; GID: 41350195	-119	541	OOF2	Sense	A
RAB1A ; GID: 41350195	-119	541	OOF2	Sense	A
RAB1A ; GID: 41350195	-119	541	OOF2	Sense	A
RAB1A ; GID: 41350195	-119	541	OOF2	Sense	A
RAB1A ; GID: 41350195	-119	541	OOF2	Sense	A
RAB1A ; GID: 41350195	-119	537	OOF2	Sense	A
RAB1A ; GID: 41350195	-119	537	OOF2	Sense	A
RAB1A ; GID: 41350195	-119	541	OOF2	Sense	A
RAB1A ; GID: 41350195	-119	541	OOF2	Sense	A
RAB1A ; GID: 41350195	-119	541	OOF2	A	Sense
RAB1A ; GID: 41350195	-119	541	OOF2	Sense	A
RAB1A ; GID: 41350195	-119	537	OOF2	Sense	A
RAB1A ; GID: 41350195	-117	538	OOF1	Sense	A
RAB1A ; GID: 41350195	-107	538	OOF2	Sense	A
RAB1A ; GID: 41350195	-107	538	OOF2	Sense	A

RAB1A ; GID: 41350195	-107	2278	OOF2	Sense	A
RAB1A ; GID: 41350195	-107	538	OOF2	Sense	A
RAB1A ; GID: 41350195	-107	538	OOF2	Sense	A
RAB1A ; GID: 41350195	-107	538	OOF2	Sense	A
RAB1A ; GID: 41350195	-107	538	OOF2	Sense	A
RAB1A ; GID: 41350195	-107	538	OOF2	Sense	A
RAB1A ; GID: 41350195	-107	538	OOF2	Sense	A
RAB1A ; GID: 41350195	-107	538	OOF2	Sense	A
RAB1A ; GID: 41350195	-107	538	OOF2	Sense	A
RAB1A ; GID: 41350195	-107	538	OOF2	Sense	A
RAB1A ; GID: 41350195	-107	538	OOF2	Sense	A
RAB1A ; GID: 41350195	-107	2278	OOF2	Sense	A
RAB1A ; GID: 41350195	-103	538	IF	A	Sense
RAB1A ; GID: 41350195	-101	579	OOF2	A	Sense
RAB1A ; GID: 41350195	-101	579	OOF2	A	Sense
RAB1A ; GID: 41350195	-101	579	OOF2	A	Sense
RAB1A ; GID: 41350195	-101	579	OOF2	A	Sense
RAB1A ; GID: 41350195	-101	579	OOF2	A	Sense
RAB1A ; GID: 41350195	-99	538	OOF1	A	Sense
RAB1A ; GID: 41350195	-99	538	OOF1	A	Sense
RAB1A ; GID: 41350195	-78	579	OOF1	A	Sense
RAB1A ; GID: 41350195	-74	538	OOF2	A	Sense
RAB1A ; GID: 41350195	-74	538	OOF2	A	Sense
RAB1A ; GID: 41350195	-74	538	OOF2	A	Sense
RAB1A ; GID: 41350195	-67	536	IF	A	Sense
RAB1A ; GID: 41350195	-67	536	IF	A	Sense
RAB1A ; GID: 41350195	-67	536	IF	A	Sense
RAB1A ; GID: 41350195	-59	538	OOF2	A	Sense
RAB1A ; GID: 41350195	-59	538	OOF2	A	Sense
RAB1A ; GID: 41350195	-59	538	OOF2	A	Sense
RAB1A ; GID: 41350195	-44	ND	OOF2	A	Sense
RAB1A ; GID: 41350195	-44	1195	OOF2	A	Sense
RAB1A ; GID: 41350195	-44	1284	OOF2	A	Sense
RAB1A ; GID: 41350195	-43	539	IF	A	Sense
RAB1A ; GID: 41350195	-32	538	OOF2	A	Sense
RAB1A ; GID: 41350195	-32	538	OOF2	A	Sense
RAB1A ; GID: 41350195	-32	538	OOF2	A	Sense
RAB1A ; GID: 41350195	-32	538	OOF2	A	Sense
RAB1A ; GID: 41350195	-32	538	OOF2	A	Sense
RAB1A ; GID: 41350195	-32	538	OOF2	A	Sense
RAB1A ; GID: 41350195	-32	538	OOF2	A	Sense
RAB1A ; GID: 41350195	-32	538	OOF2	A	Sense
RAB1A ; GID: 41350195	-32	538	OOF2	A	Sense
RAB1A ; GID: 41350195	-32	538	OOF2	A	Sense
RAB1A ; GID: 41350195	-32	538	OOF2	A	Sense
RAB1A ; GID: 41350195	-32	538	OOF2	A	Sense

RAB1A ; GID: 41350195	-32	538	OOF2	A	Sense
RAB1A ; GID: 41350195	-32	538	OOF2	A	Sense
RAB1A ; GID: 41350195	-32	538	OOF2	A	Sense
RAB1A ; GID: 41350195	-32	538	OOF2	A	Sense
RAB1A ; GID: 41350195	-32	538	OOF2	A	Sense
RAB1A ; GID: 41350195	-32	538	OOF2	A	Sense
RAB1A ; GID: 41350195	ND	536	??	A	Sense
RAB1A ; GID: 41350195	ND	537	??	A	Sense
RAB1A ; GID: 41350195	ND	537	??	A	Sense
RAB1A ; GID: 41350195	ND	537	??	A	Sense
RAB1A ; GID: 41350195	18	671	IF	A	Sense
RAB1A ; GID: 41350195	18	671	IF	A	Sense
RAB1A ; GID: 41350195	39	649	IF	A	Sense
RAB1A ; GID: 54695927	-32	538	OOF2	N/A	Sense
RAB1B ; GID: 116014337	-49	878	IF	A	Sense
RAB1B ; GID: 116014337	-49	517	IF	A	Sense
RAB1B ; GID: 116014337	-49	567	IF	A	Sense
RAB1B ; GID: 116014337	-49	878	IF	A	Sense
RAB1B ; GID: 116014337	-45	567	OOF1	A	Sense
RAB1B ; GID: 116014337	-45	567	OOF1	A	Sense
RAB1B ; GID: 116014337	-45	567	OOF1	A	Sense
RAB1B ; GID: 116014337	-45	567	OOF1	A	Sense
RAB1B ; GID: 116014337	-45	567	OOF1	A	Sense
RAB1B ; GID: 116014337	-45	567	OOF1	A	Sense
RAB1B ; GID: 116014337	-43	499	IF	A	Sense
RAB1B ; GID: 116014337	-43	499	IF	A	Sense
RAB1B ; GID: 116014337	-43	499	IF	A	Sense
RAB1B ; GID: 116014337	-43	878	IF	A	Sense
RAB1B ; GID: 116014337	-43	499	IF	A	Sense
RAB1B ; GID: 116014337	-43	499	IF	A	Sense
RAB1B ; GID: 116014337	-43	499	IF	A	Sense
RAB1B ; GID: 116014337	-43	499	IF	A	Sense
RAB1B ; GID: 116014337	-43	499	IF	A	Sense
RAB1B ; GID: 116014337	-43	499	IF	A	Sense
RAB1B ; GID: 116014337	-33	568	OOF1	A	Sense
RAB1B ; GID: 116014337	-33	568	OOF1	A	Sense
RAB1B ; GID: 116014337	-33	568	OOF1	A	Sense
RAB1B ; GID: 116014337	-33	568	OOF1	A	Sense
RAB1B ; GID: 116014337	-33	568	OOF1	A	Sense
RAB1B ; GID: 116014337	-33	568	OOF1	A	Sense
RAB1B ; GID: 116014337	-33	568	OOF1	A	Sense
RAB1B ; GID: 116014337	-22	516	IF	A	Sense
RAB1B ; GID: 116014337	-13	924	IF	A	Sense
RAB1B ; GID: 116014337	21	875	IF	A	Sense
RAB1B ; GID: 116014337	21	532	IF	A	Sense
RBM22 ; GID: 14733998	-61	679	IF	A	Sense

RBM22 ; GID: 14733998	-25	920	IF	A	Sense
RBM22 ; GID: 14733998	-16	677	IF	A	Sense
RBM22 ; GID: 14733998	-13	576	IF	A	Sense
RBM22 ; GID: 14733998	9	576	IF	A	Sense
RBM22 ; GID: 14733998	45	1101	IF	A	Sense
RBM22 ; GID: 14733998	45	1101	IF	A	Sense
RBM22 ; GID: 14733998	45	1101	IF	A	Sense
VMAC ; GID: 84992991	-35	995	OOF2	N/A	Sense
VMAC ; GID: 84992991	ND	994	??	N/A	Sense
Human - GenMatch ; GID: 4003398	-1	647	IF	B	Sense
Human - GenMatch ; GID: 4003398	90	412	IF	B	Sense
Human - GenMatch ; GID: 4003398	90	412	IF	B	Sense

ND : no data

PBS, Predicted Biological Score
