# Supplementary material for: Characterization of single chain antibody targets through yeast two hybrid
Source: BMC Biotechnol. 2010 Aug 22;10:59. doi: 10.1186/1472-6750-10-59 (PMC2936416; doi:10.1186/1472-6750-10-59)
Supplement: Additional file 4 — TA10 Two Hybrid Screen Results using human cDNA library. A table listing the identity of all the hits recovered in the two-Hybrid screen using TA10 as a bait against a human cDNA library. The table presents the name and accession number of each prey (identified by alignment, see materials and methods), the nucleotide start and stop of the insert, whether it is in frame or out of frame (OOF), its sense in the prey vector and the calculated PBS score (see materials and methods). [file 1472-6750-10-59-S4.PDF]

Additional file 4: TA10 Two Hybrid Screen Results using human cDNA library

Gene Name (Best Match)	Start	Stop	Frame	Orientation	Global PBS
hC14orf92; gi|15489161|	603	ND	IF	Sense	D
hDEDD; NM_004216.2	-123	382	OOF1	Sense	N/A
hGiantin; NM_004487.1	4137	5166	IF	Sense	A
hGiantin; NM_004487.1	4137	5166	IF	Sense	A
hGiantin; NM_004487.1	4137	5166	IF	Sense	A
hGiantin; NM_004487.1	4137	5166	IF	Sense	A
hGiantin; NM_004487.1	4180	5327	OOF1	Sense	A
hGiantin; NM_004487.1	4180	5327	OOF1	Sense	A
hGiantin; NM_004487.1	4225	4976	OOF1	Sense	A
hHMG20A; NM_018200.2	3	987	IF	Sense	E
hMBD1; NM_002384.1	-88	873	IF	Sense	D
hPIASY; NM_015897.2	-10	629	IF	Sense	E
hPIASY; NM_015897.2	6	742	IF	Sense	E
hPTP4A2; NM_003479.2	237	1078	IF	Sense	D
hREPIN1; NM_014374.1	2216	ND	??	AntiSense	N/A
hRREB1; NM_001003699.1	267	1135	IF	Sense	E
hSNX5; NM_152227.1	-103	774	IF	Sense	E
hSNX5; NM_152227.1	-97	774	IF	Sense	E
hSNX5; NM_152227.1	-97	987	IF	Sense	E
hSNX5; NM_152227.1	-97	987	IF	Sense	E
hSNX5; NM_152227.1	-97	987	IF	Sense	E
hSNX5; NM_152227.1	-97	987	IF	Sense	E
hSNX5; NM_152227.1	-97	987	IF	Sense	E
hSNX5; NM_152227.1	-55	1188	IF	Sense	E
hSNX5; NM_152227.1	-49	777	IF	Sense	E
hSNX5; NM_152227.1	-49	777	IF	Sense	E
hSNX5; NM_152227.1	-49	777	IF	Sense	E
hSNX5; NM_152227.1	-46	775	IF	Sense	E
hSNX5; NM_152227.1	-46	1022	IF	Sense	E
hSNX5; NM_152227.1	-46	775	IF	Sense	E
hSNX5; NM_152227.1	-46	775	IF	Sense	E
hSNX5; NM_152227.1	-46	940	IF	Sense	E
hSNX5; NM_152227.1	-46	1022	IF	Sense	E
hSNX5; NM_152227.1	-46	940	IF	Sense	E
hSNX5; NM_152227.1	-46	940	IF	Sense	E
hSNX5; NM_152227.1	-46	940	IF	Sense	E
hSNX5; NM_152227.1	-31	1289	IF	Sense	E
hSNX5; NM_152227.1	-31	769	IF	Sense	E
hSNX5; NM_152227.1	-31	769	IF	Sense	E
hSNX5; NM_152227.1	15	1596	IF	Sense	E
hSNX5; NM_152227.1	36	770	IF	Sense	E
hSNX5; NM_152227.1	36	770	IF	Sense	E
hSNX5; NM_152227.1	36	770	IF	Sense	E
hSNX5; NM_152227.1	36	770	IF	Sense	E
hSNX5; NM_152227.1	36	770	IF	Sense	E
hSNX5; NM_152227.1	36	770	IF	Sense	E
hSNX5; NM_152227.1	36	770	IF	Sense	E

hSNX6; NM_152233.1	6	1020	IF	Sense	E
hSNX6; NM_152233.1	6	1020	IF	Sense	E
hSWI/SNF; gi|30583190|	27	908	IF	Sense	D
hTAF1; NM_004606.2	3369	4172	IF	Sense	E
hTEAD3; NM_003214.2	39	771	IF	Sense	E
hTIMP2; NM_003255.3	1430	2217	OOF2	Sense	N/A
hZNF319; NM_020807.1	552	ND	IF	Sense	D
hZNF350; NM_021632.3	666	2028	IF	Sense	E
hZNF350; NM_021632.3	855	2060	IF	Sense	E
hHEYL; NM_014571.2	-7	641	IF	Sense	E
hHEYL; NM_014571.2	12	616	IF	Sense	E
hHEYL; NM_014571.2	12	616	IF	Sense	E
mitochondrial DNA gi|33466037|	9364	9676			N/A

ND : no data

PBS, Predicted Biological Score
