# Supplementary material for: Characterization of single chain antibody targets through yeast two hybrid
Source: BMC Biotechnol. 2010 Aug 22;10:59. doi: 10.1186/1472-6750-10-59 (PMC2936416; doi:10.1186/1472-6750-10-59)
Supplement: Additional file 5 — SF9 Two Hybrid Screen Results using human cDNA library. A table listing the identity of all the hits recovered in the two-Hybrid screen using SF9 as a bait against a human cDNA library. The table presents the name and accession number of each prey (identified by alignment, see materials and methods), the nucleotide start and stop of the insert, whether it is in frame or out of frame (OOF), its sense in the prey vector and the calculated PBS score (see materials and methods). [file 1472-6750-10-59-S5.PDF]

Additional file 5: SF9 Two Hybrid Screen Results using human cDNA library

Gene Name (Best Match)	Start	Stop	Frame	Orientation	Global PBS
hC6orf60; NM_024581.3	1925	3191	OOF2	Sense	N/A
hCKAP4; NM_006825.2	1566	2571	IF	Sense	D
hDKFZp547A023; NM_018704.1	48	1065	IF	Sense	D
hDNCH1; NM_001376.2	2744	3759	OOF2	Sense	N/A
hGLUL; gi|33877647|	-100	818	IF	Sense	A
hGLUL; gi|33877647|	-52	765	IF	Sense	A
hGLUL; gi|33877647|	-43	792	IF	Sense	A
hGLUL; gi|33877647|	-43	792	IF	Sense	A
hGLUL; gi|33877647|	-43	792	IF	Sense	A
hGLUL; gi|33877647|	-43	792	IF	Sense	A
hGLUL; gi|33877647|	-43	792	IF	Sense	A
hGLUL; gi|33877647|	-43	792	IF	Sense	A
hGLUL; gi|33877647|	-43	792	IF	Sense	A
hGLUL; gi|33877647|	-43	792	IF	Sense	A
hGLUL; gi|33877647|	-43	792	IF	Sense	A
hGLUL; gi|33877647|	-43	792	IF	Sense	A
hGLUL; gi|33877647|	-37	1041	IF	Sense	A
hGLUL; gi|33877647|	6	1041	IF	Sense	A
hGOLGB1; NM_004487.1	3843	4749	IF	Sense	B
hGOLGB1; NM_004487.1	3843	4749	IF	Sense	B
hGOLGB1; NM_004487.1	3843	4749	IF	Sense	B
hGOLGB1; NM_004487.1	4137	5166	IF	Sense	B
hGOLGB1; NM_004487.1	4137	5166	IF	Sense	B
hGOLGB1; NM_004487.1	4137	5166	IF	Sense	B
hGOLGB1; NM_004487.1	4137	5166	IF	Sense	B
hGOLGB1; NM_004487.1	4137	5166	IF	Sense	B
hGOLGB1; NM_004487.1	4137	5166	IF	Sense	B
hGOLGB1; NM_004487.1	4137	5166	IF	Sense	B
hMYH10; NM_005964.1	ND	5594	??	Sense	A
hMYH10; NM_005964.1	ND	6135	??	Sense	A
hMYH10; NM_005964.1	ND	5113	??	Sense	A
hMYH10; NM_005964.1	ND	5113	??	Sense	A
hMYH10; NM_005964.1	ND	5113	??	Sense	A
hMYH10; NM_005964.1	ND	5113	??	Sense	A
hMYH10; NM_005964.1	ND	5113	??	Sense	A
hMYH10; NM_005964.1	ND	5113	??	Sense	A
hMYH10; NM_005964.1	4201	4841	OOF1	Sense	A
hMYH10; NM_005964.1	4201	4841	OOF1	Sense	A
hMYH10; NM_005964.1	4231	5497	OOF1	Sense	A
hMYH10; NM_005964.1	4240	5365	OOF1	Sense	A
hMYH10; NM_005964.1	4240	5365	OOF1	Sense	A
hMYH10; NM_005964.1	4246	4724	OOF1	Sense	A
hMYH10; NM_005964.1	4246	4724	OOF1	Sense	A
hMYH10; NM_005964.1	4246	4724	OOF1	Sense	A
hMYH10; NM_005964.1	4246	4724	OOF1	Sense	A
hMYH10; NM_005964.1	4262	5506	OOF2	Sense	A

hMYH10; NM_005964.1	4282	5611	OOF1	Sense	A
hMYH10; NM_005964.1	4282	5611	OOF1	Sense	A
hMYH10; NM_005964.1	4356	5622	IF	Sense	A
hMYH10; NM_005964.1	4356	5622	IF	Sense	A
hMYH10; NM_005964.1	4356	5622	IF	Sense	A
hMYH10; NM_005964.1	4356	5622	IF	Sense	A
hMYH10; NM_005964.1	4356	5622	IF	Sense	A
hMYH10; NM_005964.1	4356	5622	IF	Sense	A
hMYH10; NM_005964.1	4356	5622	IF	Sense	A
hMYH10; NM_005964.1	4363	5518	OOF1	Sense	A
hMYH10; NM_005964.1	4365	5597	IF	Sense	A
hMYH10; NM_005964.1	4375	5362	OOF1	Sense	A
hMYH10; NM_005964.1	4420	6130	OOF1	Sense	A
hMYH10; NM_005964.1	4438	6134	OOF1	Sense	A
hMYH10; NM_005964.1	4462	5131	OOF1	Sense	A
hMYH10; NM_005964.1	4468	5360	OOF1	Sense	A
hMYH10; NM_005964.1	4500	4725	IF	Sense	A
hMYH10; NM_005964.1	4514	5111	OOF2	Sense	A
hMYH10; NM_005964.1	4514	5111	OOF2	Sense	A
hMYH10; NM_005964.1	4514	5111	OOF2	Sense	A
hMYH10; NM_005964.1	4587	5595	IF	Sense	A
hMYH10; NM_005964.1	4587	5595	IF	Sense	A
hMYH10; NM_005964.1	4587	5595	IF	Sense	A
hMYH10; NM_005964.1	4587	5595	IF	Sense	A
hMYH10; NM_005964.1	4587	5595	IF	Sense	A
hMYH10; NM_005964.1	4587	5595	IF	Sense	A
hMYH10; NM_005964.1	4587	5595	IF	Sense	A
hMYH10; NM_005964.1	4603	5249	OOF1	Sense	A
hMYH10; NM_005964.1	4608	ND	IF	Sense	A
hMYH10; NM_005964.1	4609	5516	OOF1	Sense	A
hMYH10; NM_005964.1	4609	5516	OOF1	Sense	A
hMYH10; NM_005964.1	4609	5516	OOF1	Sense	A
hMYH10; NM_005964.1	4612	5242	OOF1	Sense	A
hMYH10; NM_005964.1	4612	5297	OOF1	Sense	A
hMYH10; NM_005964.1	4624	5374	OOF1	Sense	A
hMYH10; NM_005964.1	4624	6136	OOF1	Sense	A
hMYH10; NM_005964.1	4627	5162	OOF1	Sense	A
hMYH11; NM_002474.1	ND	5048	??	Sense	A
hMYH11; NM_002474.1	ND	5581	??	Sense	A
hMYH11; NM_002474.1	4032	4830	IF	Sense	A
hMYH11; NM_002474.1	4131	4779	IF	Sense	A
hMYH11; NM_002474.1	4131	4779	IF	Sense	A
hMYH11; NM_002474.1	4204	5049	OOF1	Sense	A
hMYH11; NM_002474.1	4204	5049	OOF1	Sense	A
hMYH11; NM_002474.1	4204	5049	OOF1	Sense	A
hMYH11; NM_002474.1	4233	5613	IF	Sense	A
hMYH11; NM_002474.1	4233	5613	IF	Sense	A
hMYH11; NM_002474.1	4233	5613	IF	Sense	A
hMYH11; NM_002474.1	4233	5613	IF	Sense	A
hMYH11; NM_002474.1	4233	5613	IF	Sense	A

hMYH11; NM_002474.1	4234	4780	OOF1	Sense	A
hMYH11; NM_002474.1	4234	4780	OOF1	Sense	A
hMYH11; NM_002474.1	4234	4780	OOF1	Sense	A
hMYH11; NM_002474.1	4234	5045	OOF1	Sense	A
hMYH11; NM_002474.1	4234	5138	OOF1	Sense	A
hMYH11; NM_002474.1	4234	5138	OOF1	Sense	A
hMYH11; NM_002474.1	4234	5138	OOF1	Sense	A
hMYH11; NM_002474.1	4234	5138	OOF1	Sense	A
hMYH11; NM_002474.1	4234	5138	OOF1	Sense	A
hMYH11; NM_002474.1	4234	5138	OOF1	Sense	A
hMYH11; NM_002474.1	4234	5138	OOF1	Sense	A
hMYH11; NM_002474.1	4252	5582	OOF1	Sense	A
hMYH11; NM_002474.1	4255	4921	OOF1	Sense	A
hMYH11; NM_002474.1	4255	4921	OOF1	Sense	A
hMYH11; NM_002474.1	4255	4921	OOF1	Sense	A
hMYH11; NM_002474.1	4255	4921	OOF1	Sense	A
hMYH11; NM_002474.1	4255	4921	OOF1	Sense	A
hMYH11; NM_002474.1	4255	4921	OOF1	Sense	A
hMYH11; NM_002474.1	4255	4921	OOF1	Sense	A
hMYH11; NM_002474.1	4255	4921	OOF1	Sense	A
hMYH11; NM_002474.1	4255	4921	OOF1	Sense	A
hMYH11; NM_002474.1	4255	4921	OOF1	Sense	A
hMYH11; NM_002474.1	4255	4921	OOF1	Sense	A
hMYH11; NM_002474.1	4255	4921	OOF1	Sense	A
hMYH11; NM_002474.1	4255	4921	OOF1	Sense	A
hMYH11; NM_002474.1	4255	4921	OOF1	Sense	A
hMYH11; NM_002474.1	4255	4921	OOF1	Sense	A
hMYH11; NM_002474.1	4255	4921	OOF1	Sense	A
hMYH11; NM_002474.1	4255	4921	OOF1	Sense	A
hMYH11; NM_002474.1	4255	4921	OOF1	Sense	A
hMYH11; NM_002474.1	4324	5516	OOF1	Sense	A
hMYH11; NM_002474.1	4324	5516	OOF1	Sense	A
hMYH11; NM_002474.1	4327	5301	OOF1	Sense	A
hMYH11; NM_002474.1	4393	5707	OOF1	Sense	A
hMYH11; NM_002474.1	4411	5252	OOF1	Sense	A
hMYH11; NM_002474.1	4411	5252	OOF1	Sense	A
hMYH11; NM_002474.1	4411	5252	OOF1	Sense	A
hMYH11; NM_002474.1	4414	5247	OOF1	Sense	A
hMYH11; NM_002474.1	4414	5247	OOF1	Sense	A
hMYH11; NM_002474.1	4414	5247	OOF1	Sense	A
hMYH11; NM_002474.1	4414	5247	OOF1	Sense	A
hMYH11; NM_002474.1	4414	5247	OOF1	Sense	A
hMYH11; NM_002474.1	4441	5288	OOF1	Sense	A
hMYH11; NM_002474.1	4483	5097	OOF1	Sense	A
hMYH11; NM_002474.1	4483	5097	OOF1	Sense	A
hMYH11; NM_002474.1	4483	5097	OOF1	Sense	A
hMYH11; NM_002474.1	4504	5089	OOF1	Sense	A
hMYH11; NM_002474.1	4549	5614	OOF1	Sense	A
hMYH11; NM_002474.1	4569	5658	IF	Sense	A
hMYH9; NM_002473.3	ND	5707	??	Sense	A
hMYH9; NM_002473.3	ND	5479	??	Sense	A

hMYH9;	NM_002473.3	3930	4902	IF	Sense	A
hMYH9;	NM_002473.3	4053	4900	IF	Sense	A
hMYH9;	NM_002473.3	4053	4900	IF	Sense	A
hMYH9;	NM_002473.3	4090	4714	OOF1	Sense	A
hMYH9;	NM_002473.3	4119	4758	IF	Sense	A
hMYH9;	NM_002473.3	4123	4748	OOF1	Sense	A
hMYH9;	NM_002473.3	4138	4832	OOF1	Sense	A
hMYH9;	NM_002473.3	4140	4899	IF	Sense	A
hMYH9;	NM_002473.3	4147	4745	OOF1	Sense	A
hMYH9;	NM_002473.3	4147	4745	OOF1	Sense	A
hMYH9;	NM_002473.3	4161	5331	IF	Sense	A
hMYH9;	NM_002473.3	4162	5002	OOF1	Sense	A
hMYH9;	NM_002473.3	4162	5002	OOF1	Sense	A
hMYH9;	NM_002473.3	4180	4900	OOF1	Sense	A
hMYH9;	NM_002473.3	4183	5294	OOF1	Sense	A
hMYH9;	NM_002473.3	4192	5146	OOF1	Sense	A
hMYH9;	NM_002473.3	4201	4720	OOF1	Sense	A
hMYH9;	NM_002473.3	4201	4720	OOF1	Sense	A
hMYH9;	NM_002473.3	4204	5278	OOF1	Sense	A
hMYH9;	NM_002473.3	4204	5278	OOF1	Sense	A
hMYH9;	NM_002473.3	4204	5278	OOF1	Sense	A
hMYH9;	NM_002473.3	4204	5278	OOF1	Sense	A
hMYH9;	NM_002473.3	4204	5278	OOF1	Sense	A
hMYH9;	NM_002473.3	4204	5278	OOF1	Sense	A
hMYH9;	NM_002473.3	4204	5278	OOF1	Sense	A
hMYH9;	NM_002473.3	4204	5278	OOF1	Sense	A
hMYH9;	NM_002473.3	4207	4903	OOF1	Sense	A
hMYH9;	NM_002473.3	4207	4903	OOF1	Sense	A
hMYH9;	NM_002473.3	4207	4903	OOF1	Sense	A
hMYH9;	NM_002473.3	4207	4903	OOF1	Sense	A
hMYH9;	NM_002473.3	4213	4815	OOF1	Sense	A
hMYH9;	NM_002473.3	4213	4815	OOF1	Sense	A
hMYH9;	NM_002473.3	4213	5293	OOF1	Sense	A
hMYH9;	NM_002473.3	4215	5073	IF	Sense	A
hMYH9;	NM_002473.3	4215	5073	IF	Sense	A
hMYH9;	NM_002473.3	4215	5073	IF	Sense	A
hMYH9;	NM_002473.3	4216	5281	OOF1	Sense	A
hMYH9;	NM_002473.3	4216	4713	OOF1	Sense	A
hMYH9;	NM_002473.3	4216	4749	OOF1	Sense	A
hMYH9;	NM_002473.3	4216	5281	OOF1	Sense	A
hMYH9;	NM_002473.3	4216	4749	OOF1	Sense	A
hMYH9;	NM_002473.3	4216	4713	OOF1	Sense	A
hMYH9;	NM_002473.3	4216	4749	OOF1	Sense	A
hMYH9;	NM_002473.3	4219	4785	OOF1	Sense	A
hMYH9;	NM_002473.3	4234	5043	OOF1	Sense	A
hMYH9;	NM_002473.3	4234	5043	OOF1	Sense	A
hMYH9;	NM_002473.3	4234	5043	OOF1	Sense	A
hMYH9;	NM_002473.3	4234	5018	OOF1	Sense	A
hMYH9;	NM_002473.3	4234	5043	OOF1	Sense	A
hMYH9;	NM_002473.3	4234	5043	OOF1	Sense	A

hMYH9;	NM_002473.3	4234	5018	OOF1	Sense	A
hMYH9;	NM_002473.3	4234	5018	OOF1	Sense	A
hMYH9;	NM_002473.3	4234	5043	OOF1	Sense	A
hMYH9;	NM_002473.3	4234	5018	OOF1	Sense	A
hMYH9;	NM_002473.3	4234	5043	OOF1	Sense	A
hMYH9;	NM_002473.3	4234	5043	OOF1	Sense	A
hMYH9;	NM_002473.3	4242	4902	IF	Sense	A
hMYH9;	NM_002473.3	4252	4900	OOF1	Sense	A
hMYH9;	NM_002473.3	4252	4900	OOF1	Sense	A
hMYH9;	NM_002473.3	4252	4900	OOF1	Sense	A
hMYH9;	NM_002473.3	4252	4900	OOF1	Sense	A
hMYH9;	NM_002473.3	4252	4900	OOF1	Sense	A
hMYH9;	NM_002473.3	4252	4900	OOF1	Sense	A
hMYH9;	NM_002473.3	4252	4900	OOF1	Sense	A
hMYH9;	NM_002473.3	4258	4756	OOF1	Sense	A
hMYH9;	NM_002473.3	4262	5708	OOF2	Sense	A
hMYH9;	NM_002473.3	4264	4793	OOF1	Sense	A
hMYH9;	NM_002473.3	4264	4793	OOF1	Sense	A
hMYH9;	NM_002473.3	4264	5951	OOF1	Sense	A
hMYH9;	NM_002473.3	4264	4793	OOF1	Sense	A
hMYH9;	NM_002473.3	4282	5110	OOF1	Sense	A
hMYH9;	NM_002473.3	4284	5166	IF	Sense	A
hMYH9;	NM_002473.3	4284	5166	IF	Sense	A
hMYH9;	NM_002473.3	4285	4805	OOF1	Sense	A
hMYH9;	NM_002473.3	4303	5251	OOF1	Sense	A
hMYH9;	NM_002473.3	4325	5020	OOF2	Sense	A
hMYH9;	NM_002473.3	4331	4873	OOF2	Sense	A
hMYH9;	NM_002473.3	4333	5021	OOF1	Sense	A
hMYH9;	NM_002473.3	4342	5296	OOF1	Sense	A
hMYH9;	NM_002473.3	4348	5042	OOF1	Sense	A
hMYH9;	NM_002473.3	4348	5042	OOF1	Sense	A
hMYH9;	NM_002473.3	4348	5590	OOF1	Sense	A
hMYH9;	NM_002473.3	4352	5480	OOF2	Sense	A
hMYH9;	NM_002473.3	4352	5480	OOF2	Sense	A
hMYH9;	NM_002473.3	4381	4975	OOF1	Sense	A
hMYH9;	NM_002473.3	4381	4975	OOF1	Sense	A
hMYH9;	NM_002473.3	4381	4975	OOF1	Sense	A
hMYH9;	NM_002473.3	4384	5023	OOF1	Sense	A
hMYH9;	NM_002473.3	4384	5023	OOF1	Sense	A
hMYH9;	NM_002473.3	4384	5023	OOF1	Sense	A
hMYH9;	NM_002473.3	4384	5023	OOF1	Sense	A
hMYH9;	NM_002473.3	4384	5023	OOF1	Sense	A
hMYH9;	NM_002473.3	4384	5023	OOF1	Sense	A
hMYH9;	NM_002473.3	4388	5296	OOF2	Sense	A
hMYH9;	NM_002473.3	4390	5488	OOF1	Sense	A
hMYH9;	NM_002473.3	4390	5488	OOF1	Sense	A
hMYH9;	NM_002473.3	4390	4970	OOF1	Sense	A
hMYH9;	NM_002473.3	4393	5176	OOF1	Sense	A
hMYH9;	NM_002473.3	4393	5176	OOF1	Sense	A
hMYH9;	NM_002473.3	4393	5176	OOF1	Sense	A

hMYH9;	NM_002473.3	4393	5176	OOF1	Sense	A
hMYH9;	NM_002473.3	4393	4871	OOF1	Sense	A
hMYH9;	NM_002473.3	4393	5569	OOF1	Sense	A
hMYH9;	NM_002473.3	4393	5569	OOF1	Sense	A
hMYH9;	NM_002473.3	4393	4871	OOF1	Sense	A
hMYH9;	NM_002473.3	4393	4871	OOF1	Sense	A
hMYH9;	NM_002473.3	4393	4871	OOF1	Sense	A
hMYH9;	NM_002473.3	4393	4835	OOF1	Sense	A
hMYH9;	NM_002473.3	4393	5569	OOF1	Sense	A
hMYH9;	NM_002473.3	4393	4871	OOF1	Sense	A
hMYH9;	NM_002473.3	4393	5296	OOF1	Sense	A
hMYH9;	NM_002473.3	4393	5569	OOF1	Sense	A
hMYH9;	NM_002473.3	4393	5569	OOF1	Sense	A
hMYH9;	NM_002473.3	4393	5569	OOF1	Sense	A
hMYH9;	NM_002473.3	4402	5711	OOF1	Sense	A
hMYH9;	NM_002473.3	4402	5018	OOF1	Sense	A
hMYH9;	NM_002473.3	4402	5018	OOF1	Sense	A
hMYH9;	NM_002473.3	4402	5711	OOF1	Sense	A
hMYH9;	NM_002473.3	4402	5711	OOF1	Sense	A
hMYH9;	NM_002473.3	4405	5398	OOF1	Sense	A
hMYH9;	NM_002473.3	4411	5144	OOF1	Sense	A
hMYH9;	NM_002473.3	4411	5144	OOF1	Sense	A
hMYH9;	NM_002473.3	4426	4806	OOF1	Sense	A
hMYH9;	NM_002473.3	4427	4805	OOF2	Sense	A
hMYH9;	NM_002473.3	4432	5522	OOF1	Sense	A
hMYH9;	NM_002473.3	4432	5522	OOF1	Sense	A
hMYH9;	NM_002473.3	4435	5293	OOF1	Sense	A
hMYH9;	NM_002473.3	4435	5293	OOF1	Sense	A
hMYH9;	NM_002473.3	4435	5293	OOF1	Sense	A
hMYH9;	NM_002473.3	4435	5293	OOF1	Sense	A
hMYH9;	NM_002473.3	4435	5293	OOF1	Sense	A
hMYH9;	NM_002473.3	4441	5188	OOF1	Sense	A
hMYH9;	NM_002473.3	4444	5238	OOF1	Sense	A
hMYH9;	NM_002473.3	4444	5238	OOF1	Sense	A
hMYH9;	NM_002473.3	4447	5289	OOF1	Sense	A
hMYH9;	NM_002473.3	4447	5289	OOF1	Sense	A
hMYH9;	NM_002473.3	4447	5017	OOF1	Sense	A
hMYH9;	NM_002473.3	4447	5017	OOF1	Sense	A
hMYH9;	NM_002473.3	4462	5225	OOF1	Sense	A
hMYH9;	NM_002473.3	4462	5225	OOF1	Sense	A
hMYH9;	NM_002473.3	4462	5225	OOF1	Sense	A
hMYH9;	NM_002473.3	4462	5225	OOF1	Sense	A
hMYH9;	NM_002473.3	4462	5225	OOF1	Sense	A
hMYH9;	NM_002473.3	4462	5225	OOF1	Sense	A
hMYH9;	NM_002473.3	4462	5225	OOF1	Sense	A
hMYH9;	NM_002473.3	4462	5225	OOF1	Sense	A
hMYH9;	NM_002473.3	4462	5225	OOF1	Sense	A
hMYH9;	NM_002473.3	4462	5225	OOF1	Sense	A
hMYH9;	NM_002473.3	4462	5225	OOF1	Sense	A
hMYH9;	NM_002473.3	4462	5225	OOF1	Sense	A

hMYH9; NM_002473.3	4471	5043	OOF1	Sense	A
hMYH9; NM_002473.3	4471	5043	OOF1	Sense	A
hMYH9; NM_002473.3	4476	6365	IF	Sense	A
hMYH9; NM_002473.3	4477	5567	OOF1	Sense	A
hMYH9; NM_002473.3	4482	5013	IF	Sense	A
hMYH9; NM_002473.3	4482	5013	IF	Sense	A
hMYH9; NM_002473.3	4482	4915	IF	Sense	A
hMYH9; NM_002473.3	4489	5171	OOF1	Sense	A
hMYH9; NM_002473.3	4489	5171	OOF1	Sense	A
hMYH9; NM_002473.3	4507	5269	OOF1	Sense	A
hMYH9; NM_002473.3	4507	5269	OOF1	Sense	A
hMYH9; NM_002473.3	4507	5269	OOF1	Sense	A
hMYH9; NM_002473.3	4528	4975	OOF1	Sense	A
hMYH9; NM_002473.3	4528	4975	OOF1	Sense	A
hMYH9; NM_002473.3	4537	5296	OOF1	Sense	A
hMYH9; NM_002473.3	4537	5296	OOF1	Sense	A
hMYH9; NM_002473.3	4546	ND	OOF1	Sense	A
hMYH9; NM_002473.3	4546	5480	OOF1	Sense	A
hMYH9; NM_002473.3	4549	5477	OOF1	Sense	A
hMYH9; NM_002473.3	4570	5143	OOF1	Sense	A
hMYH9; NM_002473.3	4572	5319	IF	Sense	A
hMYH9; NM_002473.3	4572	5319	IF	Sense	A
hMYH9; NM_002473.3	4588	5761	OOF1	Sense	A
hMYH9; NM_002473.3	4600	5567	OOF1	Sense	A
hMYH9; NM_002473.3	4600	5564	OOF1	Sense	A
hMYH9; NM_002473.3	4600	5566	OOF1	Sense	A
hMYH9; NM_002473.3	4600	5566	OOF1	Sense	A
hMYH9; NM_002473.3	4600	5566	OOF1	Sense	A
hMYH9; NM_002473.3	4600	5566	OOF1	Sense	A
hMYH9; NM_002473.3	4600	5566	OOF1	Sense	A
hMYH9; NM_002473.3	4600	5566	OOF1	Sense	A
hMYH9; NM_002473.3	4600	5567	OOF1	Sense	A
hMYH9; NM_002473.3	4600	5564	OOF1	Sense	A
hMYH9; NM_002473.3	4600	5567	OOF1	Sense	A
hMYH9; NM_002473.3	4600	5567	OOF1	Sense	A
hMYH9; NM_002473.3	4600	5566	OOF1	Sense	A
hMYH9; NM_002473.3	4600	5020	OOF1	Sense	A
hMYH9; NM_002473.3	4603	5329	OOF1	Sense	A
hMYH9; NM_002473.3	4603	5329	OOF1	Sense	A
hMYH9; NM_002473.3	4603	5329	OOF1	Sense	A
hMYH9; NM_002473.3	4603	5329	OOF1	Sense	A
hMYH9; NM_002473.3	4606	5250	OOF1	Sense	A
hMYH9; NM_002473.3	4606	5594	OOF1	Sense	A
hMYH9; NM_002473.3	4611	5319	IF	Sense	A
hRCBTB1; NM_018191.3	953	1948	OOF2	Sense	N/A
hRCBTB1; NM_018191.3	953	1948	OOF2	Sense	N/A
hSEC10L1; NM_006544.2	93	381	IF	Sense	D
hSEC10L1; NM_006544.2	93	381	IF	Sense	D
hSLC9A1; NM_003047.2	1606	1144	??	AntiSense	N/A
hSNRP70; [gi|12654934|	-28	462	IF	Sense	E

hTGM2; NM_004613.2	2986	3237	OOF1	Sense	N/A
hTGM2; NM_004613.2	2986	3237	OOF1	Sense	N/A
hUnknown gi|33875002|	-208	201	IF	Sense	D
gi|30582690|	503	ND	??	AntiSense	N/A
Mitochondrial DNA	6075	6745	IF	Sense	N/A
unknown; GenMatch]	-1	510	IF	Sense	D
unknown; GenMatch	-1	510	IF	Sense	D

ND : no data

PBS, Predicted Biological Score
